# The Impact of Virtual Reality on Chronic Pain

**DOI:** 10.1371/journal.pone.0167523

**Published:** 2016-12-20

**Authors:** Ted Jones, Todd Moore, James Choo

**Affiliations:** 1 Pain Consultants of East Tennessee, Knoxville, Tennessee, United States of America; 2 Psychology Department, University of Tennessee, Knoxville, Tennessee, United States of America; Tokai Daigaku, JAPAN

## Abstract

The treatment of chronic pain could benefit from additional non-opioid interventions. Virtual reality (VR) has been shown to be effective in decreasing pain for procedural or acute pain but to date there have been few studies on its use in chronic pain. The present study was an investigation of the impact of a virtual reality application for chronic pain. Thirty (30) participants with various chronic pain conditions were offered a five-minute session using a virtual reality application called Cool! Participants were asked about their pain using a 0–10 visual analog scale rating before the VR session, during the session and immediately after the session. They were also asked about immersion into the VR world and about possible side effects. Pain was reduced from pre-session to post-session by 33%. Pain was reduced from pre-session during the VR session by 60%. These changes were both statistically significant at the p < .001 level. Three participants (10%) reported no change between pre and post pain ratings. Ten participants (33%) reported complete pain relief while doing the virtual reality session. All participants (100%) reported a decrease in pain to some degree between pre-session pain and during-session pain. The virtual reality experience was found here to provide a significant amount of pain relief. A head mounted display (HMD) was used with all subjects and no discomfort was experienced. Only one participant noted any side effects. VR seems to have promise as a non-opioid treatment for chronic pain and further investigation is warranted.

## Introduction

Many Americans suffer from chronic pain and much of that pain is under-treated or treated ineffectively [1–3]. Meanwhile, misuse and diversion of opioids used to treat chronic pain conditions have increased dramatically in recent years [4]. This leaves those who treat chronic pain conditions with the twin dilemma of working to treat chronic pain conditions in an effective manner while prescribing opioid medications in a very cautious manner, if prescribed at all. Safe, effective alternatives to opioid medications are vitally important to consider and test to assist with the great number of persons with chronic pain conditions who need to be helped without the concerns related to the use of opioid medications.

Virtual reality (VR) has been shown to be an effective adjunct or alternative to opioid analgesics even in cases of high levels of pain such as burn pain and wound care [5–15]. These numerous studies have demonstrated that VR is an effective tool in significantly reducing pain in acute pain situations. The “gate theory” of attention is the most widely accepted model in explaining the impact of VR on pain [16, 17]. Gate theory of attention postulates that VR reduces the perception of pain by absorbing and diverting attention away from the pain.

Some authors have opined that VR could be an effective tool in treating chronic pain conditions. Keefe et al state that while there is growing evidence supporting VR’s effectiveness in managing acute procedural pain, little is known about the use of VR for chronic pain [18]. However, chronic pain is known to be substantially different from acute pain [19]. Multiple variables are involved with the sensation of chronic pain, including various psychological factors and central nervous system processes. It is possible that virtual reality, while having been shown to be effective for acute pain, is ineffective for chronic pain due to these factors.

A review of the current literature finds that there has been only one study to date assessing the impact of VR on chronic pain [20]. In this study participants were asked about their pain before a VR session and while they were in the VR session. The session was 15 minutes long and used pleasant and relaxing scenes. The study did not investigate the impact of VR on chronic pain after the VR session was over. One past study conducted in Israel investigated the impact of VR on itching caused by atopic dermatitis and psoriasis vulgaris. In this study data were gathered on the impact during a VR session and ten minutes afterwards [21]. This study found that VR significantly decreased itching during the VR sessions and also at ten minutes afterwards. Interestingly, a video game was also effective in decreasing itching during the session but did not decrease itching afterwards.

It is currently unknown whether VR has an impact on chronic pain after a VR session, though there is reason to think that it would based on the above study on itching. If VR can be shown to have an impact on the experience of chronic pain after a VR session is over, this could open a number of possibilities for the use of VR for chronic pain syndromes. The purpose of this present study was to determine the impact of a brief VR session on the experience of pain in patients with chronic pain conditions, assessing the impact both during and immediately following the session. This study also assessed the issue of whether chronic pain patients can tolerate the VR session without the side effects that sometimes come with VR such as headaches, dizziness or nausea.

## Methods

The study was approved by the Institutional Review Board (IRB) of the University of Tennessee Knoxville. Participants were recruited from an outpatient pain practice in Knoxville, Tennessee (Pain Consultants of East Tennessee). Recruitment flyers were placed in the practice lobby and in the physical therapy area. Participation in the study was voluntary and had no bearing on the patient’s pain treatment. To qualify for the study, participants had to be at least 18 years old, must not be visually or hearing impaired, had to be an active patient at the pain practice, had to have had an initial psychological assessment, and had to have been assessed at the initial psychological assessment as having sufficient cognitive faculties to give informed consent. Participants who meet the eligibility criteria and agreed to be in the study were given the informed consent form to sign. After their written informed consent was obtained, a time was arranged for the virtual reality experience. This was usually immediately following the signing of the informed consent form.

Using a sample size calculator for a paired t test, we assumed a .05 level of statistical probability and a power of .8. One recent article [22] conducted a meta-analysis on studies to date which had investigated the use of VR for acute or experimentally-induced pain. Based on these data we assumed an effect size of .5 and a standard deviation of pre-post change scores of 1.0. These choices and assumptions yielded a sample size of 31. Rounding this figure we chose a sample size of 30 for the study.

The primary outcome measure used on this study was a 0–10 numerical rating scale of pain. A 0–10 numerical rating of pain has been shown to be a valid and reliable method of rating pain intensity and shows better compliance, responsiveness and ease of use compared to verbal rating scales or visual analog scales [23]. The anchors for the numerical rating scale were “Ten is the worst pain anyone could ever have and zero is no pain at all.”

**Fig 1:**
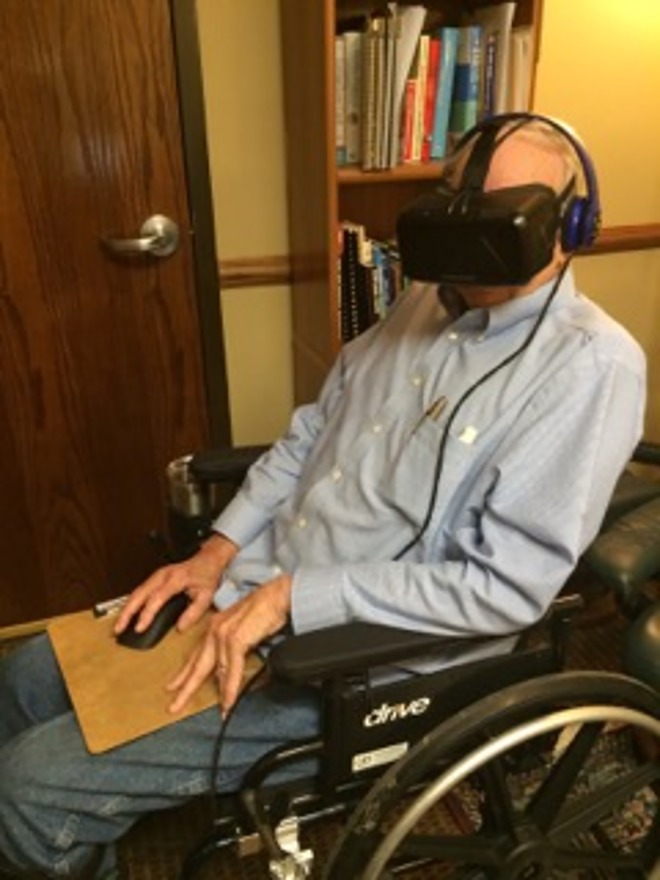
Photo of participant using the virtual reality application Cool!.

When the VR session occurred, the equipment and the general visual experience were explained to the participant and all questions were answered. Before the VR session started he or she was asked what his or her pain level was on the 0–10 numerical rating scale. The participant donned the virtual reality headset, headphones placed on the subject and he or she was given a mouse on a clipboard that was placed in his or her lap. The participant then engaged in the virtual reality experience (described below) for five minutes. Fig 1 below shows what the virtual reality setup and activity looks like to the outside observer. After the five minute experience the VR equipment was removed and the participant was asked what his or her pain was on the 0–10 numerical rating scale at that moment. The participant was then asked what his or her pain level was during the virtual reality experience, again using the numerical rating scale.

Participants were further asked two questions about engagement in the virtual reality experience: “On a scale of 0–10, to what extent did you feel like you went inside the virtual world? Ten is ‘I went completely inside the virtual world’ and 0 is ‘I did not feel like I went inside it at all’ and also: “On a scale of 0–10, how real did the objects seem to you? Ten is ‘indistinguishable from the real world’ and 0 is ‘completely fake.’” Participants were also asked three questions about any side effects “On a scale of 0–10 how much dizziness did you feel while you were in the virtual world? Ten is ‘feel faint’ and zero is ‘no dizziness at all’”, and “On a scale of 0–10 how bad a headache did you feel while you were in the virtual world? Ten is ‘worst headaches possible’ and zero is ‘no headache at all’ and “On a scale of 0–10 how much nausea did you feel while you were in the virtual world? Ten is ‘vomited’ and zero is ‘no nausea at all’”. This ended the session. A cash reward of five dollars was given to all participants after session completion and was not dependent on their responses. The intervention then was a single five-minute exposure to the virtual reality application with a primary outcome measure of one numerical pain rating scale score obtained for pre-session, post-session and during the VR session.

**Fig 2:**
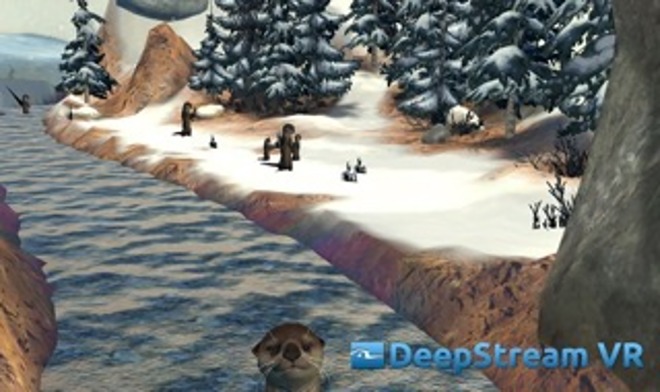
Screen shot from Cool! showing otters.

**Fig 3:**
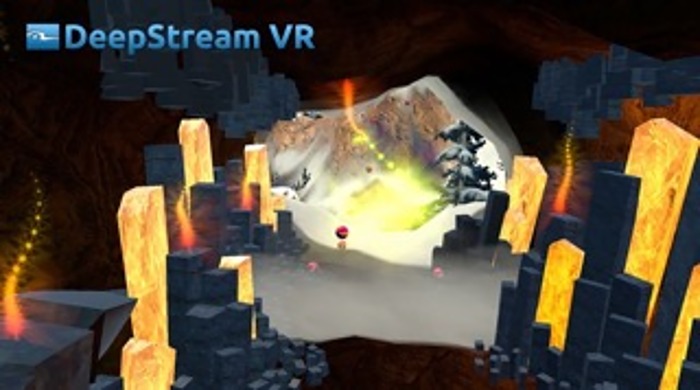
Screen shot from Cool! showing caves and flames.

The VR application used is these studies is called COOL! (DeepStream VR Inc., 2014). COOL! is an interactive journey through a fully immersive 360° VR fantasy landscape. Participants are taken along a route through a virtual landscape (see Figs 2 and 3 for screen shots). Objects include trees, hills, snow scenes, caves, flames and otters. The speed is slow and constant and can not be manipulated by the participant. There is ambient music delivered by the application through headphones. The music is moderate in volume and participants would still hear the experimenter and converse as much as they desired. Once started the participant “travels” through the landscape at a constant speed until the application automatically stops at the time limit (five minutes). The scene is 360° and participants can look forward, left, right, above, below and behind them in a seamless manner. Participants can interact with various aspects of the landscape as they “travel” using the buttons of a mouse. Right clicks will toss orbs and left clicks will toss fish. When hit, flames will make sounds and change colors. When hit, otters will move about in a playful way and change colors. There is no violence involved. There is no score to be kept and participants were told they could toss as many orbs or fish as they liked or none at all. Participants were assured that they could stop any time for any reason but none did and all participants had the full five minute experience, with several participants asking if they could continue longer.

There are two ways that participants could view Cool!. One was with a head-mounted display (HMD) using an Oculus Rift DK2. This was the default method of viewing Cool!. One past study has shown that HMD’s extensive facial contact points are particularly problematic with head injuries, allodynia or claustrophobia [11]. To ensure participants’ comfort if the participant has facial pain or for any reason was uncomfortable with the HMD, an alternate method for viewing Cool! was available. This alternate viewer, the DeepStream 3D Viewer (DS), is a stereoscopic 3D display which uses conventional technologies to achieve high immersion. A previous study comparing the two technologies (HMD versus the DeepStream 3D Viewer) has found no difference between the two in pain reduction or immersion effect [24].

## Results

Thirty (30) participants were recruited to the study. Sixty-seven (67) percent were female, The median age was 50 years old with a range of 35 to 79 years. Ninety-six percent were Caucasian. Participants had a variety of chronic pain disorders, including cervical spine pain (9), lumbar spine pain (6), hip pain (6), pervasive pain from myalgia or connective tissue disease (2), interstitial cystitis (2), chest wall pain (1), shoulder pain (1), abdominal pain (1), thoracic spinal pain (1), and neuropathy (1). The average reported duration of their pain was 16 years, with the range being 1 year to 43 years. All participants then met the definition of having a chronic pain disorder.

The average pre-session pain rating for the 30 participants was 5.7. The average post-session pain rating was 4.1 and the during-session average pain rating was 2.6. The average change in pain rating between pre-session ratings and post-session ratings was -1.6 and resulted in a 33% reduction in pain. A paired t-test of pre-post session ratings found this change to be significant at the p < .001 level. The average change in pain rating between pre-session ratings and during-session ratings was –3.1 and resulted in a 60% reduction in pain. A paired t-test also found this change to be significant at the p < .001 level. Three participants (10%) reported no change between pre and post pain ratings. All participants (100%) reported a decrease in pain to some degree between pre-session pain and during-session pain. Ten participants (33%) reported 100% pain relief while doing the virtual reality session.

Participants were asked to rate their engagement in the virtual world 0–10. The average rating on this item was 8.4. The participants were asked how real the virtual world seemed to them 0–10. The average rating on this item was 6.5. As to side effects participants were asked about dizziness, headaches and nausea, rating each 0–10. No participant complained about any degree of dizziness or headache with the HMD. One participant rated nausea as a 3/10. She noted that she had a long history of nausea with video games and said that this application was better than the video games she has used before in not triggering nausea. All other participants reported no degree of nausea. All participants used the Oculus Rift DK2 to view Cool! and the DeepStream 3D Viewer was never used or needed to be offered due to discomfort.

## Summary, Discussion and Limitations

This study set out to determine the impact of a brief virtual reality experience on the experience of chronic pain. Participants were given a five-minute experience of the virtual reality application called Cool!. Data was gathered on the level of chronic pain felt immediately before the session, during the session and immediately after the session. The data obtained here found that a five-minute virtual reality experience decreased the sensation of chronic pain by an average of 33% from pre-session and to post-session. Participants reported an average decrease in pain of 60% between pre-session and during the experience. Both of these decreases in pain sensation were significant at the p < .001 level. Participants generally reported that they felt involved in the experience and that it seemed fairly “real” to them. Only one participant reported any side effect of any kind. This finding is in contrast to the previous study on VR with chronic pain which showed that participants had some side effects though at low levels [20].

An HMD device was used (Oculus Rift DK2). An alternative viewer from a HMD device was available for use if side effects were present or if there was discomfort from the HMD but no participant had trouble with or discomfort from the HMD. This was despite the fact that several participants had a diagnosis of neck or head pain. Thus, unlike one past study which found some discomfort with an HMD [11], this was not a problem here.

Spontaneous comments about the VR experience included the following. “I absolutely love this. Oh my God, this thing is so cool. That was amazing”; “I’d like to do more hours of it, not just because of the benefits but because it was fun”; “I thoroughly enjoyed it. I would like to do the longer version”; “It was relaxing. It would be good for depression”; and “Wow, I had no toothache while I was doing that.” One participant wanted the ability to change speeds during the experience and one complained that the images were “grainy.” One said there was a “frustration factor” as he could not hit the otters consistently with orbs or fish.

It is interesting to note that the median age was 50 years old. Chronic pain patients are often somewhat familiar with technology but are generally older than a younger often technologically savvy population. Still, this older population had no problems using or enjoying this technology. It is also interesting to note that when used on an Oculus Rift the application Cool! requires participants to aim their throws by turning their head to look directly at an object. The VR experience was well-received by all participants despite the fact that the most common pain disorder among participants was cervical spine pain. Apparently participants were involved or distracted enough that no one complained about having to turn their head to throw fish or orbs.

The virtual reality experience was found here to provide a significant amount of pain relief and the analgesia was present immediately after the session was over. As a measure of comparison for analgesia, one meta-analysis found that morphine reduces pain by about 30% [25]. The analgesia experienced by participants here was comparable to morphine between pre-session and post-session and was almost double that when comparing between pre-session and during-session pain ratings. Decreasing the use of opioid analgesics and using alternatives to opioids in treating chronic pain has been a clarion call recently [26]. The data found here support continued investigation of virtual reality as a treatment for chronic pain.

There are several limitations to this study. There was no comparison group in this study. Comparing this VR intervention with other methods of reducing pain such guided relaxation or distraction could help clarify the specific mechanisms and relative power of this intervention for chronic pain. A review by Li et al (2011) notes that while VR has been shown to offer improved analgesia over other types of distractions (such as cartoons and video games) the exact mechanisms that produce the analgesia are as yet unknown [27]. The selective and focused attention that is elicited during VR appears to be supplemented by emotional and cognitive factors as well. The richness of the sensory and cognitive experience in VR appears to produce more impactful results that more simple and unisensory forms of distraction. Additional research is needed to parse out which factors are most important for patients with chronic pain.

One potential confounder to the results found here is that the principle investigator was conducting the VR session and collecting the outcome data. It could be that experimenter bias was introduced through this process. A future study in which the VR session is conducted by an unaffiliated person could assess the presence of this bias. Additionally, the participants in this VR were aware of the study purposes and could have been biased to expect an effect. Finally, another possible confounder was the way in which the participants were obtained. Participants were recruited through flyers at a pain clinic and self-selected to participate. It is likely that those who chose to be in the study were motivated and had expectations about potential pain relief. A future study could involve participants who, while voluntarily agreeing to be in the study, are not recruited through a voluntary selection process.

There are a host of questions to be addressed in future research. As above, more studies are needed comparing VR with other pain reduction techniques. These studies should help determine what elements of this intervention are most important and central to its effect. Another question is about the duration of the analgesia from VR. It was found here that chronic pain levels were reduced after a VR session. What is not known is how long the duration of analgesia might be. We anecdotally heard some participants in this study report that their analgesia lasted for hours or even days after the VR session was over. This leads back to the question about the underlying neural mechanisms of analgesia of VR. The effectiveness of VR could be based solely on attentional factors through the gate theory mechanisms or it could be that endorphin release (pleasure of the game and experiencing pain relief) could be playing a role as well. The answers to these questions could help determine the future relative emphasis that could be placed on the development of VR systems that can be used at home on a ongoing basis or whether office-based systems might be preferred.

Another question for further study is whether longer VR sessions could produce greater analgesia over time. This study used a five-minute VR session. It is possible that longer sessions can produce analgesia that has significant duration, which is particularly important for chronic pain conditions. Also, which VR applications produce more or less analgesia is an empirical question that should be studied. Likely there are certain characteristics of VR applications that can enhance analgesia and these need to be explored.

Gender and age differences are important variables to be investigated in future studies. It may well be that gender and age are tied to the effects or lack of effects found in the use of virtual reality for pain. Similarly, chronic pain is a heterogeneous disease. It may be that VR is more effective for chronic pain disorders that more intimately involve the central nervous system such as CRPS or fibromyalgia while being less effective for more mechanically-based pain disorders such as low back pain or arthritic conditions. Moving forward, studies will need to parse these variables out to determine for which chronic pain patients VR might be particularly effective.

The present study found that, in this sample, the majority of chronic pain patients experienced significant analgesia with a brief VR session. We found very few side effects to be associated with the experience. VR appears to have some promise as a non-opioid treatment for chronic pain and further investigation appears warranted.

## Supporting Information

S1 File (XLSX)

## References

[1] Federation of State Medical Boards (FSMB): Model Policy on the Use of Opioid Analgesics in the Treatment of Chronic Pain. Washington, DC: The Federation, 2013.

[2] Noble M, Treadwell JR, Tregear SJ, Coates VH, Wiffen PJ, Akafomo C, et al *Cochrane Database of Systematic Reviews*, *Issue 1* Long-term Opioid Management for Chronic Noncancer Pain. New York, NY: The Cochrane Collaborative, John Wiley & Sons, Ltd, 2010. 10.1002/14651858.CD006605.pub2 PMC6494200 20091598

[3] Rosenblum A, Marsch LA, Joseph H, Portnoy RK. 2008 Opioids and the treatment of chronic pain: Controversies, current status, and future directions. Exp Clin Psychopharm. 16 (5): 405–416. 10.1037/a0013628 PMC2711509 18837637

[4] Office of National Drug Control Policy (ONDCP): Epidemic: Responding to America's Prescription Drug Abuse Crisis. Washington, DC: Executive Office of the President, The White House, 2011.

[5] Gershon J, Zimand E, Pickering M, Rothbaum BO, Hodges L. 2004 A pilot and feasibility study of virtual reality as a distraction for children with cancer. Jrnl Amer Acad Chld Adol Psychtry, 43(10), 1243–9. 10.1097/01.chi.0000135621.23145.05 15381891

[6] Hoffman HG, Patterson DR, Carrougher GJ. 2000 Use of virtual reality for adjunctive treatment of adult burn pain during physical therapy: a controlled study. Clin Jrnl Pain, 16(3), 244–250. 10.1097/00002508-200009000-00010 11014398

[7] Hoffman HG, Patterson DR, Carrougher GJ, Sharar SR. 2001 Effectiveness of virtual reality-based pain control with multiple treatments. Clin Jrnl Pain, 17(3), 229–35. Retrieved from http://www.ncbi.nlm.nih.gov/pubmed/11587113 10.1097/00002508-200109000-00007 11587113

[8] Hoffman HG, Patterson DR, Seibel E, Soltani M, Jewett-Leahy L, Sharar SR. 2008 Virtual reality pain control during burn wound debridement in the hydrotank. Clin Jrnl Pain, 24(4), 299–304. 10.1097/AJP.0b013e318164d2cc 18427228

[9] Chan EA, Chung JW, Wong TK, Lien AS, & Yang JY. (2007). Application of a virtual reality prototype for pain relief of pediatric burn in Taiwan. Jrnl Clin Nrsng, 16(4), 786–93. 10.1111/j.1365-2702.2006.01719.x 17402961

[10] Maani CV, Hoffman HG, DeSocio PA, Morrow M, Gaylin C, Magula J, et al 2008 Pain control during wound care for combat-related burn injuries using custom articulated arm mounted virtual reality goggles. Jrnl Cybrthrpy Rehab, 1(2), 193.

[11] Maani CV, Hoffman HG, Morrow M, Maiers A, Gaylord K, McGhee LL, et al 2011 Virtual reality pain control during burn wound debridement of combat-related burn injuries using robot-like arm mounted VR goggles. Jrnl Traum, 71(1 Suppl), S125–30. 10.1097/TA.0b013e31822192e2 PMC4460976 21795888

[12] Mosso JL, Obrador GT, Wiederhold B, Wiederhold M, Lara V, Santander A. 2012 Cybertherapy in Medicine–Experience at the Universidad Panamericana, IMSS and ISSSTE Mexico.

[13] Patterson DR, Hoffman HG, Palacios AG, Jensen MJ. 2006 Analgesic effects of posthypnotic suggestions and virtual reality distraction on thermal pain. Jrnl Abnrml Psych, 115, 834–841. 10.1037/0021-843X.115.4.834 17100541

[14] Sharar SR, Carrougher GJ, Nakamura D, Hoffman HG, Blough DK, Patterson DR. 2007 Factors influencing the efficacy of virtual reality distraction analgesia during postburn physical therapy: preliminary results from 3 ongoing studies. Arch Phys Med Rehab, 88(12 Suppl 2), S43–9. 10.1016/j.apmr.2007.09.004 18036981

[15] Van Twillert B, Bremer M, Faber AW. 2007 Computer-generated virtual reality to control pain and anxiety in pediatric and adult burn patients during wound dressing changes. Jrnl Burn Care Resch, 28(5), 694–702. 10.1097/BCR.0B013E318148C96F 17667488

[16] Melzack R, Wall PD. 1965 Pain assessment, a new theory. Science, 150, 971–5. 5320816 10.1126/science.150.3699.971

[17] Gold JI, Belmont K, Thomas D. 2007 The neurobiology of virtual reality pain attenuation. Cyberpsy Beh, The Impact of the Internet, Multimedia and Virtual Reality on Behavior and Society, 10(4), 536–44. 10.1089/cpb.2007.9993 17711362

[18] Keefe FJ, Huling DA, Coggins MJ, Keefe DF, Rosenthal MZ, Herr NR, et al Virtual Reality for Persistent Pain: A New Direction for Behavioral Pain Management. 2012 Pain. 153 (11):2163–2166. 10.1016/j.pain.2012.05.030 22770840 PMC3472118

[19] Grichnik KP, Ferrante FM. The Difference Between Acute and Chronic Pain. Mt. Sinai J Med. 1991 5 58(3):217–20. 1875958

[20] Wiederhold BK, Gao K, Sulea C, Wiederhold MD. 2014 Virtual Reality as a Distraction Technique in Chronic Pain Patients. Cyberpsy, Beh Soc Net. 17(6):346–352. 10.1089/cyber.2014.0207 PMC4043365 24892196

[21] Leibovici V, Magora F, Cohen S, Ingber A. 2009 Effects of Virtual Reality Immersion and Audiovisual Distraction Techniques for Patients with Pruritus. Pain Res Manage 14(4):283–286. 10.1155/2009/178751 PMC2734514 19714267

[22] Kenny MP, Milling LS. 2016. The Effectiveness of Virtual Reality Distraction for Reducing Pain: A Meta-analysis. Psychology of Consciousness: Theory, Research and Practice. Advance online publication.

[23] Hjermstad MJ, Fayers PM, Haugen DF, Caraceni A, Hanks GW, Loge JH, et al 2011 Studies comparing Numerical Rating Scales, Verbal Rating Scales, and Visual Analogue Scales for assessment of pain intensity in adults: a systematic review. J Pain Symptom Manage.41(6):1073–93. 10.1016/j.jpainsymman.2010.08.016 21621130

[24] Pickrell JE, Hollander A, Mancl L, Rose H, Drangsholt MT, Gromala D, et al. 2014. Taking the Helmet off of Virtual Reality: Efficacy of an Arm-Mounted Virtual Reality Display for Reduction of Heat Pain. Unpublished manuscript.

[25] Furlan AD, Sandoval JA, Mailis-Gagnon A, Tunks E. 2006 Opioids for chronic noncancer pain: a meta-analysis of effectiveness and side effects. CMAJ;174(11):1589–94. 10.1503/cmaj.051528 16717269 PMC1459894

[26] Dowell D, Haegerich TM, Chou R. 2016 CDC guideline for prescribing opioids for chronic pain United States, 2016. MMWR Recomm Rep. 65:1–49. 10.15585/mmwr.rr6501e1 26987082

[27] Li A, Montaño Z, Chen VJ, Gold JI. 2011 Virtual reality and pain management: current trends and future directions. Pain Management, 1(2), 147–157. 10.2217/pmt.10.15 21779307 PMC3138477

